# Targeting Breast Cancer and Their Stem Cell Population through AMPK Activation: Novel Insights

**DOI:** 10.3390/cells11030576

**Published:** 2022-02-07

**Authors:** Bhawna Uprety, Heidi Abrahamse

**Affiliations:** Laser Research Centre, Faculty of Health Sciences, University of Johannesburg, P.O. Box 17011, Doornfontein 2028, South Africa; habrahamse@uj.ac.za

**Keywords:** AMPK activation, cancer stem cells, non-oncology drugs, vanadium

## Abstract

Despite some significant advancements, breast cancer has become the most prevalent cancer in the world. One of the main reasons for failure in treatment and metastasis has been attributed to the presence of cancer initiating cells—cancer stem cells. Consequently, research is now being focussed on targeting cancer cells along with their stem cell population. Non-oncology drugs are gaining increasing attention for their potent anticancer activities. Metformin, a drug commonly used to treat type 2 diabetes, is the best example in this regard. It exerts its therapeutic action by activating 5′ adenosine monophosphate-activated protein kinase (AMPK). Activated AMPK subsequently phosphorylates and targets several cellular pathways involved in cell growth and proliferation and the maintenance of stem-like properties of cancer stem cells. Therefore, AMPK is emerging as a target of choice for developing effective anticancer drugs. Vanadium compounds are well-known PTP inhibitors and AMPK activators. They find extensive applications in treatment of diabetes and obesity via PTP1B inhibition and AMPK-mediated inhibition of adipogenesis. However, their role in targeting cancer stem cells has not been explored yet. This review is an attempt to establish the applications of insulin mimetic vanadium compounds for the treatment of breast cancer by AMPK activation and PTP1B inhibition pathways.

## 1. Introduction

Breast cancer is the most common cancer in women of all races across the globe. According to the World Health Organization, 2.3 million women were diagnosed with breast cancer in 2020 alone, with approximately 685,000 deaths reported globally. As per the 2021 WHO statistics, breast cancer has become the most prevalent cancer in the world, and it is estimated that it would constitute over 30% of all cancers diagnosed in women [1,2]. However, recent advances in medicine and technology have caused significant improvement in the early detection and treatment of cancer through radiation and targeted chemotherapy using specific surface markers (ALDH1, CD44) on cancer and cancer stem cells [3]. Targeted chemotherapies, such as immunotherapy and metal–drug nanoconjugates, have been developed based on gene expression profiling and cancer subtype as well as hormonal therapies for hormone receptor-positive subtype cancers and many more. However, although these approaches cause an initial positive response, there remains a high risk for tumour relapse and drug resistance. In many cases, the relapsed tumour metastasizes, and currently remains incurable [4]. Recent studies suggest that most of these drawbacks experienced with the current line of treatment are caused by a minute population of cells within cancer cells—cancer stem cells (CSCs). The occurrence of cancer stem cells amongst breast cancer cells (BCCs) was first established by Al-Hajj et al. They discovered that as few as 100 of these cells were potent enough to form tumours when xenotransplanted in mice [5].

**Figure 1:**
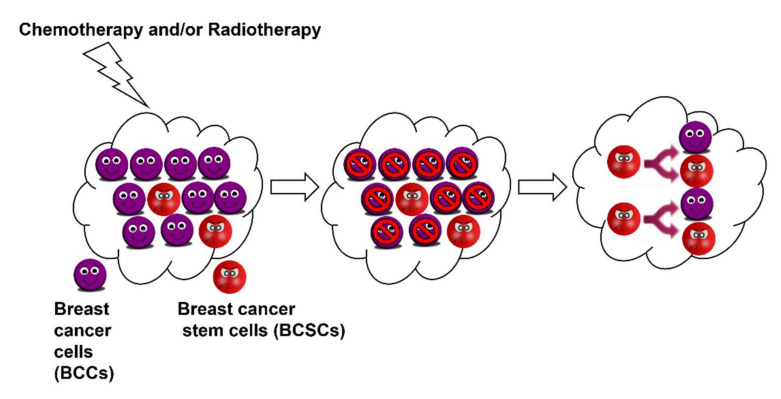
Breast cancer stem cells, or cancer-initiating cells, display an innate ability to evade and survive all treatment strategies owing to their stem-like properties. They function to maintain the cancer cell population while regenerating themselves continuously.

Cancer stem cells are capable of asymmetric cell division: self-renewal and the generation of heterogeneous lineages (Figure 1). Breast cancer stem cells have been shown to differentiate into endothelial cells to support vasculogenic mimicry and angiogenesis, which subsequently provides nutritional supplies and oxygen to support propagating tumour cells. They are also known to be more resistant to treatment and programmed cell death (anoikis). These sturdy and strong cells can migrate through blood circulation thus causing metastasis. Therefore, it is imperative to kill breast cancer stem cells along with breast cancer cells to ensure complete treatment and prevention of relapse and metastasis. Consequently, BCSCs have emerged as important therapeutic targets especially in overcoming metastasis and drug resistance [6]. Therefore, it is crucial to develop novel chemotherapeutics that can target and/or kill BCSCs.

### Breast Cancer Stem Cells

The term cancer stem cells essentially refer to the relatively smaller cell population within cancer cells that can repopulate the tumour cells over a long period of time as well as retain their ability to regenerate themselves continuously. Thus, cancer stem cells, or tumour-initiating cells, function like normal stem cells but in a diseased manner [7]. Pioneering work on BCSC isolation and characterisation was conducted by Hajj et al. They isolated BCSCs as CD44^+^CD24^−^/Lineage^−^ subpopulation within breast cancer cells and demonstrated that their tumorigenic ability resonated with normal stem cells. These cells can undergo self-renewal as well as differentiation. They can multiply extensively, as few as 200 of these unpassaged cells were able to form solid tumours that could be planted in mice. Their multiplication resulted in a heterogeneous population of cells composed of the CD44^+^CD24^−^/Lineage^−^ population as well as non-tumorigenic cells that could compose the bulk of the tumour mass [5].

Breast cancer stem cells display increased cell motility and invasion and they overexpress genes that promote metastasis [8]. Most cancer therapies developed and being used today were designed to target cancer cells. Cancer stem cells can easily evade these by virtue of their “stemness”. BCSCs are characterised by an overexpression of the BCL-2 family of proteins and ABC transporters as well as multiple drug resistance. They possess the inherent ability to efflux any administered drug and, thus, can elude all therapies. Therefore, BCSCs are fundamentally more resistant to the currently employed clinical techniques (chemo and radiation) [5,9]. In fact, there are a few reports suggesting that most of these treatments enhance the fraction of CD44^+^CD24^−^ cells and ALDH^+^ cells, i.e., BCSCs within the tumour cells. Therefore, it is pertinent to treat breast cancer stem cells along with breast cancer cells to ensure a complete and effective treatment. Consequently, many treatment strategies are being designed to directly target the cancer stem cell population by modulating specific pathways involved in the cancer stem cell phenotype [10,11,12]. Significant progress has been achieved in targeting the Notch pathway, Wnt, and Hedgehog signalling pathway in ongoing clinical trials. In vitro studies using breast cancer stem cell markers reveal that BCSCs are relatively resistant to radiation therapy and chemotherapy. This was also resonated in clinical studies, wherein molecular profiles of tumours treated with chemotherapy closely resembled the gene profiling of CD44^+^CD24^−^ cells [13]. This resistance is imparted by stem cell specific pathways such as Wnt/β–catenin, Notch, and Hedgehog signalling. These pathways are often dysregulated in breast cancer and, thus, various approaches are being designed to target them for effective treatment [11].

The most noteworthy progress in targeted breast cancer treatment involves the development of HER2 targeted therapies using trastuzumab and lapatinib. This treatment approach is also proposed to act against the ALDH1 cancer stem cell population [14,15]. Apart from immunotherapy, novel treatment strategies are also being developed to target stem cell specific pathways. The most noteworthy of these include a combination of docetaxel and γ-secretase inhibitor MK-0752 against the Notch pathway and is currently in Phase I/II clinical trials. Similarly, clinical trials have also been initiated using combination therapies targeting the canonical Wnt signalling pathway (ClinicalTrials.gov Identifier: NCT01351103) as well as Hedgehog signalling pathways (ClinicalTrials.gov Identifier: NCT02694224).

## 2. Drug Repurposing: The Anti-Diabetic to Anticancer Action of Metformin

The discovery and understanding of the crucial role played by breast cancer stem cells in cancer propagation and metastasis has now led researchers to develop therapeutic strategies to target and treat cancerous stem cells. Several therapies have been tested in vivo and in vitro to target BCSCs through membrane markers and transporters as well as alteration of the cancer stem cell microenvironment. However, although some of these treatments have been promising in vitro, in vivo toxicity and pharmacokinetics remains a big challenge. Intriguingly, some success has been reported recently, with drugs used clinically to treat other ailments. There are researchers and clinicians all over the globe working towards finding a safe and effective cure for cancer. The average cost for the development of an anticancer drug is over 600 million dollars and takes approximately 10–20 years. Moreover, less than 1% of the drug candidates enter clinical trials, and the proportion of drugs successfully making it to market is even smaller. Therefore, drug repurposing is increasingly being employed particularly in cancer research [16,17,18]. Drug repurposing means exploring the possibility of new therapeutic applications of FDA approved drugs that were not listed in the original drug medication. The major benefit provided by drug repurposing is the fact that the said drugs have already been FDA approved and their safety and biocompatibility, i.e., pharmacokinetics and pharmacodynamics, for humans has already been established. Therefore, these drugs can move fast between preclinical and clinical trials. However, with the plethora of FDA approved drugs available, the discovery of anticancer applications of non-oncology drugs are usually serendipitous [19,20]. Recently, there was also a study reporting a systematic screening of over 4000 FDA approved drugs for over 500 cancer cell lines using PRISM software analysis [19].

Many independent systematic studies reported lower risk and occurrences of cancer in diabetic patients on metformin treatment [21,22]. Consequently, the most noteworthy results for breast cancer using a non-oncology drug have been reported with metformin [23,24,25,26,27].

Metformin acts on mitochondria in the cell, inhibiting the mitochondrial electron transport system by enhancing the AMP-activated protein kinase (AMPK) activity. 5′ adenosine monophosphate-activated protein kinase is an enzyme involved in cellular energy homeostasis, largely to activate glucose and fatty acid uptake and oxidation when cellular energy is low. AMPK is identified as the crucial candidate causing the interaction between metabolism and cancer [28,29]. Activated AMPK induces the resting catabolic state of the cell, thus restoring homeostasis. AMPK activity is also implicated in targeted breast cancer stem cells therapy. There are several reports suggesting that augmented AMPK activity eliminates breast cancer stem cells [30,31,32]. Moreover, there are numerous reports suggesting that metformin can specifically target the cancer stem cell population in breast, pancreatic, glioblastoma, and ovarian cancer models. Metformin treatment significantly reduces the sphere-forming ability and inhibits proliferation in the CD44^+^CD24^−^ and ALDH^+^ stem-cell-rich population in breast cancer cells [21,24]. Consequently, metformin is part of several ongoing clinical trials, alone and in combination with clinically used anticancer drugs such as doxorubicin and paclitaxel to target cancer stem cell population along with tumour suppression for breast cancer. The therapeutic actions of metformin are known to proceed via the induction of cellular stress by the inhibition of oxidative phosphorylation in mitochondria, followed by the activation of AMPK [29].

## 3. AMPK Activation

### Mechanism of AMPK Activation

**Figure 2:**
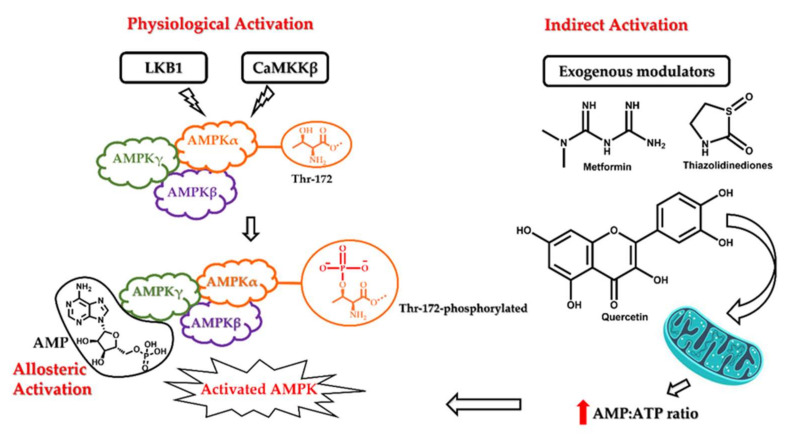
Mechanism of AMPK activation. AMPK can be activated via the LKB1 or CaMKKβ pathway (physiological activation) following phosphorylation at threonine 172 residue in the α subunit. It can also be activated exogenously by drugs such as quercetin and metformin that act on the mitochondria and increase the AMP:ATP ratio, which subsequently activates the LKB1-mediated phosphorylation and activation of AMPK. Lastly, it can also be activated allosterically by the binding of AMP at the AMPKγ subunit.

5′ adenosine monophosphate-activated protein kinase (AMPK) is an enzyme complex that undergoes autoactivation under conditions of cellular stress such as exercise, hypoxia, and hypoglycaemia. Some anti-diabetic drugs, such as metformin, and thiazolidinediones, such as pioglitazone, also exert their therapeutic effect by activating AMPK [33]. Structurally, it is a heterotrimeric protein kinase consisting of AMPK α, β, and γ subunits. The catalytic α subunit is primarily involved in the activation of the kinase, while the AMPKβ and AMPKγ subunits are mainly regulatory [34]. The generally accepted mechanism for physiological AMPK activation involves phosphorylation of the Thr-172 residue within the catalytic active site of AMPKα subunit mainly by LKB1 (liver kinase B1) and CaMKKβ (Ca^2+^/calmodulin-dependent protein kinase β) [35,36]. The LKB1 mediated Thr phosphorylation can also be AMP dependent. Under conditions of cellular stress and a high AMP:ATP ratio, AMP allosterically activates AMPK by binding to the AMPKγ and protects the physiological integrity of the activated AMPK by inhibiting its dephosphorylation. In contrast, the CaMKKβ catalysed activation of AMPK is arbitrated by the concentration of cellular calcium ions. AMPK can also be activated passively by molecules that can induce the accumulation of intracellular AMP or calcium ions. The most well-known examples of indirect activators of AMPK are anti-diabetic drugs such as metformin and pioglitazone. They act on the mitochondria and increase the AMP:ATP ratio, thereby leading to cellular stress and AMPK activation. The various mechanisms of AMPK activation are depicted in Figure 2. After activation, AMPK phosphorylates multiple substrates, initiating a cascade of cellular responses to limit ATP consumption. The overall effect of this regulation is to reduce the synthesis of cholesterol, fatty acids, ribosomal RNAs (rRNAs), and proteins. Consequently, several energy-intense pathways, such as fatty acid synthesis and oxidation, are inhibited. Activated AMPK arrests the G1 phase of the cell cycle along with the expression of apoptotic proteins, p53 and p21, and is known to induce autophagy [37,38,39,40].

## 4. AMPK Activation for the Treatment of Breast Cancer Stem Cells

AMPK is fast emerging as a target of choice in the treatment of cancer cells and cancer stem cells. It is an energy-sensitive molecule and is activated under conditions of cellular stress, a common phenomenon in cancer cells. However, a few reports argue this activation may actually protect cancer cells [41]; nevertheless, there is ample evidence to suggest the therapeutic action of AMPK activation on cancer stem cells, particularly breast cancer stem cells [31,42,43,44,45,46,47]. Paclitaxel, a well-known and commonly used drug for the treatment of breast and lung cancers, involves the activation of AMPK during its therapeutic pathway [48]. Activated AMPK also influences several cancer stem cell specific pathways, indicating the role of AMPK as a tumour suppressor [49]. Activated AMPK interrupts the G1-S and G2-M phases of mitosis, resulting in cell cycle arrest via downregulation of mTORC1 activity and reduced lipogenesis. AMPK activation can also affect the cell cycle by non-metabolic pathways. Prolonged AMPK activation also results in mitotic spindle misorientation as well as abnormal chromosome segregation [50]. Moreover, AMPK activation has also been shown to target cancer stem cell population by counteracting the epithelial-to-mesenchymal transition (EMT) in breast and prostate cancer cells by activating forkhead box O3 (Foxo3a). EMT in cancer cells is known to generate and maintain stem-like properties in cancer cells and lead to chemoresistance and metastasis [51]. Several direct and indirect AMPK activators have been proposed and tested for their anticancer activities. A-769662, a direct AMPK activator, was shown to delay tumour formation in hypomorphic mice and reduced proliferation in breast, colon and prostate cancer cells. Another AMPK activator, OSU-53, suppresses tumour growth in triple-negative breast cancer models in vitro and in vivo. OSU-53 also constrains EMT in breast and prostate cells by activating Foxo3a, thus influencing the cancer stem cell population as well [52]. Similar results have also been observed in clinical studies, wherein enhanced AMPK expression is associated with relapse-free, longer survival rates in breast cancer patients [53].

An increased AMPK activity could assist in anticancer activity through the interplay of various pathways, some of which are discussed below.

### 4.1. Activated AMPK Downregulates Cyclin Proteins and Induces Cell Cycle Arrest and Autophagy

About 30–60% of breast cancers are characterised by an overexpression of the cyclin D1 encoding gene, CCND1, and over 50% of breast cancers overexpress cyclin D1 protein. Cyclin D1 plays a crucial role in tumour progression and metastasis by causing abnormal phosphorylation and inhibiting the tumour suppressor protein, retinoblastoma (pRB) [54]. It also serves to enrich the population of cancer stem cells and induce angiogenesis. Cyclin D1 is a vital modulator of mammary and embryonic stem cells and is known to assist transforming growth factor β (TGF-β) in inducing stem cell renewal in triple-negative breast cancer cells [55,56]. Therefore, many promising studies have focussed on downregulating cyclin D1 for enhanced anticancer action [57,58,59,60]. Activated AMPK phosphorylates several substrates and different cyclin(s) leading to autophagy and cell cycle arrest. For instance, AMPK activation is a requirement for cyclin D1 downregulation [61,62]. Activated AMPK phosphorylates phosphoinositide 3-kinase enhancer-activating Akt (PIKE–A) at the Ser 351 and Ser 377 residues, thereby inducing the translocation of PIKE-A into the nucleus. Within the nucleus, PIKE-A complexes with CDK4, destabilizing the CDK4/CyclinD1 complex, thus inhibiting the cyclin D1 activity and inducing cell cycle arrest and decreased cell proliferation [63]. The LKB1–AMPK complex also catalyses the phosphorylation of p27Kip1 at the Thr 198, thereby activating the cyclin-dependent kinase inhibitor [64,65]. Furthermore, activated AMPK induces autophagy by phosphorylating S326 of cyclin Y, leading to the formation of the cyclin Y–CDK16 complex and activation of CDK16 [66]. Although the role of autophagy in cancer can be dual and conflicting, there are shreds of evidence suggesting AMPK activation-mediated autophagy results in decreased cell proliferation and cell death [67,68].

**Figure 3:**
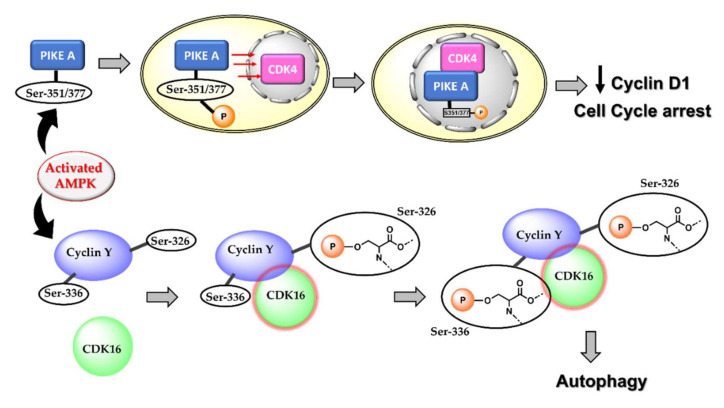
Known mechanisms of activated AMPK-mediated downregulation of cyclin D1, cell cycle arrest, and autophagy. AMPK phosphorylation activates various cyclin-dependent kinases (CDKs) to result in downregulation of cyclin D1 and cell cycle arrest in the G1/S phase as well autophagy, all of which have been implicated in anticancer therapy.

An important consequence of AMPK-mediated cyclin D1 inhibition is the arrest of cell cycle progression in the G0/G1 phase. It is also a commonly encountered pathway of cell death relating to the activation of AMPK. Many studies using metformin on different cancer cell lines as well as cancer stem cells suggest the arresting of the cell cycle at the G1:S boundary, following AMPK-mediated upregulation of CDK-inhibiting p21 and p27 [30,61,69,70,71,72]. The pathways for AMPK-mediated downregulation of cyclin D and induction of autophagy are shown in Figure 3.

### 4.2. AMPK Activation Inhibits Lipogenic Enzymes

AMPK is the master regulator of energy molecules in the body and is involved in glucose and fat metabolism. Phosphorylation of AMPK is a crucial step involved in the inhibition of adipogenesis and lipogenesis [73,74,75]. Fatty acids play crucial roles in maintaining cellular functions and structure. Normal cells exhibit low activity of fatty acid synthase enzyme; however, the activity is elevated by many folds in fast proliferating cancer cells, making it a suitable therapeutic target. Furthermore, most breast cancers exhibit raised levels of acetyl-CoA carboxylase (ACC), a key enzyme for the synthesis of long-chain fatty acids. AMPK activation downregulates the synthesis of fatty acids, lipids, triglycerides, and cholesterol by acting on multiple substrates. Activated AMPK restrains fatty acid synthesis by deactivating ACC. It affects triglyceride synthesis by inhibiting the activity of glycerol-3-phosphate acyltransferase (GPAT). It also downregulates 3-hydroxy-3-methylglutaryl-CoA reductase (HMG-CoA), a crucial enzyme involved in the synthesis of cholesterol. There are also a few studies suggesting the role of AMPK in deactivating lipogenic gene transcription factors. Therefore, it can be said that activated AMPK acts as the vital controller of lipogenic pathways; and this has direct consequences on oncogenic signalling [76]. Unlike normal cells, cancer cells are commonly characterised by enhanced activity of fatty acid and lipid synthesizing enzymes to compensate for the additional energy and nutrition requirement of fast proliferating cells [77]. These differential adipogenesis and lipogenesis activities have been targeted to develop effective and targeted anticancer therapies. Since AMPK is the master regulator of metabolism, most of these therapies are known to act via direct or indirect activation of AMPK [78,79,80,81,82].

### 4.3. AMPK Activation Downregulates the Mammalian Target of the Rapamycin (mTOR) Pathway and Insulin Growth Factors (IGFs)

**Figure 4:**
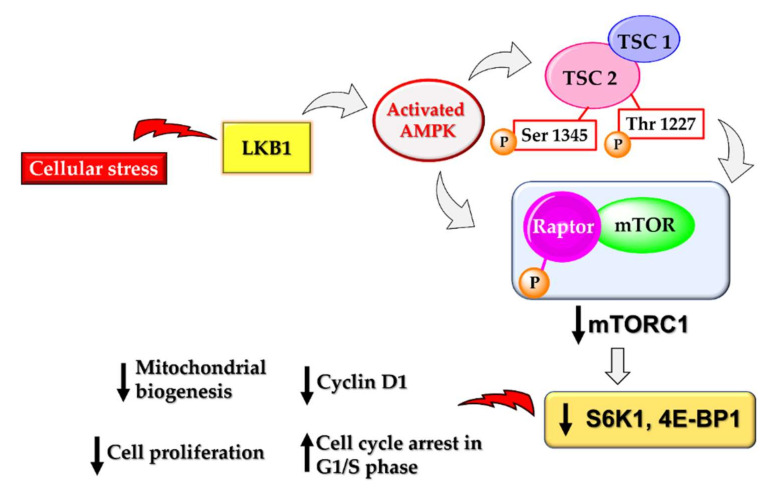
Activated AMPK-mediated downregulation of the mTORC1 complex can follow two pathways: through the phosphorylation and activation of tuberous sclerosis complex 2 (TSC 2) or via direct phosphorylation of raptor at S722 and S792. The deactivation of mTOR is subsequently followed by the inhibition of S6K1 and 4E-BP1, both of which are responsible for the initiation of protein translation and maintenance of cell size and growth.

AMPK activation also leads to the downregulation of the mechanistic target of rapamycin complex 1 (mTORC1) to restrict the ATP-expensive anabolic processes. mTORC1 is a nutrition-sensitive protein complex composed of mTOR kinase and the scaffolding protein raptor, catalysing various biosynthetic pathways in the human body [83]. mTOR is a Ser/Thr protein kinase that plays crucial roles in cell growth and survival, autophagy, cell division and proliferation. It promotes tumour growth and progression by providing nutrients to cancer cells and promotes angiogenesis. Thus, mTOR plays a pivotal role in cancer cells, particularly cancer stem cells [84]. There is increasing evidence suggesting the pivotal role played by phosphatidylinositol-3-kinase (PI3K)/Akt and the mammalian target of rapamycin (mTOR) (PI3K/Akt/mTOR) signalling in cancer stem cell biology. mTOR activity is frequently associated with the maintenance of cancer stem cells, and particularly in breast cancer stem cells, mTOR is required for tumorigenicity. mTOR along with IGF also controls cell size, growth, and proliferation in tumours [85]. Many drugs being used clinically today to treat cancers are PI3K/Akt/mTOR suppressors [86,87]. There are many studies reporting a direct link between AMPK activation and mTOR suppression [88]. Activated AMPK suppresses mTOR by phosphorylating and activating the tuberous sclerosis complex (TSC) that negatively regulates mTOR signalling [23,89,90]. It can also directly cause the phosphorylation of raptor at Ser 722 and Ser 792, thereby deactivating the activity of mTORC1 [83]. Deactivation of mTOR subsequently downregulates several downstream substrates, resulting in the loss of cell proliferation, cell shrinkage, loss of translation, and many more effects that result in apoptosis in cancer cells (Figure 4). AMPK activation by metformin also involves downregulation of mTOR for the anticancer effect [91,92]. However, there are a few reports suggesting the metformin-mediated repression of mTOR can be independent of AMPK as well [26].

### 4.4. AMPK Activity Opposes the Warburg Effect

Cancer cells are fast growing and proliferate at an uncontrolled rate. Therefore, they have higher energy requirements than normal cells. The greater energy demands are met by the Warburg effect—cancer cells that operate at higher rates of glucose uptake and lactate production irrespective of the available oxygen concentration and normal mitochondrial function [93]. The exact role of the Warburg effect on cancer cells is still a topic of research; nevertheless, it is considered an important hallmark of cancer. Furthermore, increased glucose uptake and lactate production might result in the acidosis of the tumour microenvironment. LKB1-mediated AMPK activation has been associated as a negative regulator of the Warburg effect in cancer cells [94,95,96]. Thus, AMPK is identified as the crucial candidate causing the interaction between metabolism and cancer [28]. AMPK activity also exerts anti-inflammatory properties and maintains cellular redox balance. Moreover, AMPK also increases inhibitory phosphorylation of glycogen synthase kinase 3 beta (GSK3β), which contributes to mitochondrial protection against iron-induced oxidant stress [97,98].

### 4.5. Anticancer Stem Cell Action of AMPK

Apart from the various tumour suppression mechanisms described above, activated AMPK also inhibits several self-renewal and metastasis-related pathways that are often associated with the cancer stem cell population. There are ample reports suggesting the involvement of Wnt/β-catenin, NF-κB, TNFα, interleukin proteins, Notch, and Hedgehog signalling pathways in the differentiation and proliferation of breast cancer stem cells by inducing epithelial-to-mesenchymal transition (EMT) and tumorigenesis [99]. AMPK activation results in a cascade of cellular responses that can affect the cancer stem cell phenotype in different cancers including breast cancer. Activated AMPK is known to inhibit EMT in pancreatic and breast cancer cells by downregulating the sonic Hedgehog signalling pathway and TGFβ cytokines by phosphorylating and destabilizing the transcriptional activator form of Gli-1 [29,100]. AMPK activation also inhibits the canonical Wnt signalling pathway in breast and prostate cancers by downregulating DVL3 [101,102]. The canonical and noncanonical Wnt signalling promotes stemness in breast cancer stem cells. It plays crucial roles in the proliferation, orientation, migration, and resistance in the BCSC subpopulation. Therefore, treatments targeting the Wnt/β-catenin pathway may be potent for removing BCSCs [99]. Furthermore, activated AMPK negatively impacts several inflammatory pathways, such as NF-κB, TNFα, and interleukin proteins, that play crucial roles in populating the cancer stem cell population. There are also studies suggesting the role of AMPK activators in targeting specific microRNA-mediated pathways against cancer stem cells. Metformin upregulates let-7a in MCF7 human breast cancer cells and inhibits TGFβ-induced miR-181a expression subsequently reducing the mammosphere-forming ability in vitro [103].

**Figure 5:**
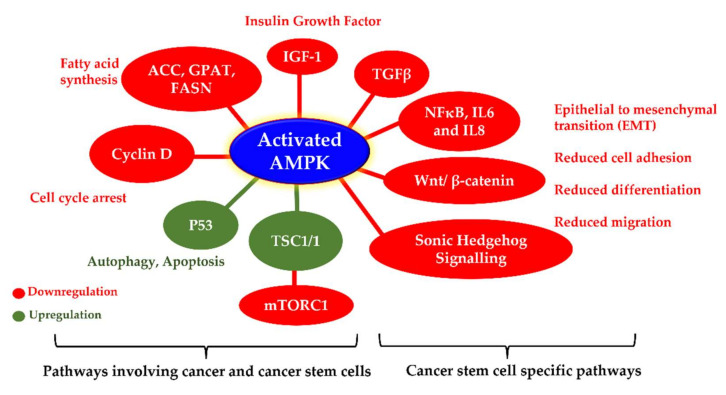
Cellular pathways associated with AMPK activation in cancer and cancer stem cells. The figure depicts various important molecular pathways mediated by AMPK activation that play pivotal roles against cancer and cancer stem cells.

Thus, AMPK is a suitable target for developing effective cancer treatments (Figure 5). Some recently reported AMPK activators with anticancer activity are compiled in Table 1.

## 5. Vanadium Compounds: Insulin Mimetic Agents with Possible Anticancer Action

### 5.1. Anti-Diabetic Action of Vanadium—PTP1B Inhibition

The anti-diabetic effects of vanadium compounds are well documented in the literature [106,107,108,109]. There are surplus reports of vanadium (IV) and (V) complexes exhibiting moderate to excellent insulin mimetic activity [106,107,110,111,112,113]. The geometrical and physiological similarity between vanadate and phosphate allows for the involvement of the former in phosphate-dependent pathways [114,115]. Vanadium compounds, particularly in oxidation states (IV) and (V), are biocompatible and have shown to improve glucose homeostasis and insulin resistance in both in vitro and in vivo models [110,111,116]. The most successful vanadium compounds to be studied with insulin mimetic properties are BMOV (bis(maltolato)oxovanadium(IV)) and its derivatives such as BEOV (bis(ethylmaltolato)oxovanadium(IV)). They have been shown to induce normoglycemic effects in streptozotocin (STZ)-induced type 1 and type 2 diabetic rats in a dose-dependent manner in the range of 1–10 mg V kg^−1^ body weight [112]. Despite their exemplary activities, these vanadium compounds have not been successful yet in pharma markets. Nevertheless, a deep understanding of their mechanism of action can be applied to cater to the needs of pharmacological and medicinal research.

**Figure 6:**
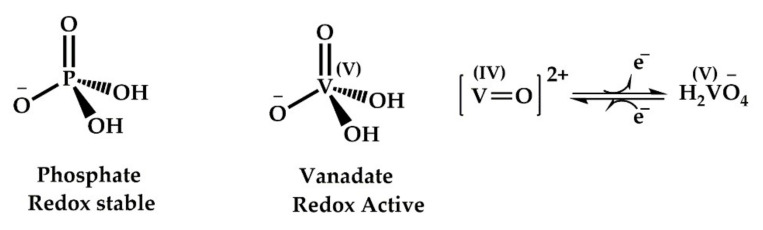
Structural similarity between phosphate and vanadate at physiological pH and the redox conversion between V (IV) and V (V) oxidation states.

Vanadium compounds are known inhibitors of several ATPases and phosphatases with varying potency [111]. The most extensively studied phosphatases in this regard have been tyrosine protein phosphatases (PTPases), alkaline and acid phosphatases. PTPases are a superfamily of enzymes that control signal transduction pathways and regulate cellular functions. They play important roless in several oncogenic and metabolic disorders [117,118]. The process of physiological phosphorylation of proteins is strictly balanced and monitored by two opposing enzymes—protein tyrosine kinases (PTKs) and protein tyrosine phosphatases. PTPases have cysteine residues at the active site, and they catalyse the hydrolysis of phosphate esters. Vanadium compounds can oxidise these cysteine residues via free radical and ROS mechanisms thus inhibiting the biological responses that depend on the thiol-reducing ability. Alkaline phosphatases are membrane-bound glycoproteins that catalyse the phosphorylation of small organic molecules. The vanadium induced inhibition of alkaline phosphatases results from the geometrical similarity between vanadates and phosphates (Figure 6). The greater coordination flexibility of vanadate allows them to mimic and replace the penta-coordinated transition states of phosphates in biological systems, thereby initiating a cascade of cellular responses and inhibition [119]. Moreover, the “hard” nature of vanadium allows for the formation of strong hydrogen bonds with amino acid residues, thereby leading to the stabilisation of negatively charged transition states in physiological processes. This also results in inactivation or inhibition of the respective enzyme and the cellular pathway. Thus, vanadium compounds can inhibit most phosphatases in a non-specific manner [114]. The insulin mimicking action of vanadium results from this inhibition, particularly the inhibition of PTP1B. PTP1B is the key negative regulator of insulin signalling [120]. The insulin receptors are present as a transmembrane protein on all mammalian cells. Insulin binding results in the phosphorylation of the tyrosine residues at the insulin receptors. This tyrosine phosphorylation subsequently activates intracellular signalling, leading to glucose uptake and metabolism. PTP1B negatively impacts the cascade by dephosphorylating the tyrosine residues at the insulin receptors. Therefore, inhibition of PTP1B improves insulin sensitivity and has been widely used to treat diabetes [121]. There are ample reviews on vanadium-mediated PTP1B inhibition [110,114,122,123], and to keep this work concise, it will not be discussed in detail here.

### 5.2. PTP1B and Breast Cancer

PTP1B is localised in the endoplasmic reticulum in the cells and downregulates leptin receptors and insulin signalling by dephosphorylating the IRS [124]. This dephosphorylation results in the deactivation of the insulin receptor, thereby leading to hyperglycaemic conditions and insulin resistance. Therefore, PTP1B has emerged as a target of choice for developing effective anti-diabetic and anti-obesity treatments [125]. The role of PTP1B in breast cancer can be contentious; it has been shown to be a suppressor as well as an oncogene. While there are a few reports suggesting the prognostic action of PTP1B in cancers [126,127], there are several reports that counteract and suggest otherwise [128,129,130]. The gene encoding for PTP1B, PTPN1, is located on chromosome number 20q13, which is often augmented in breast cancers [128], and it is overexpressed in at least 70% of all breast cancers [131,132]. PTP1B expression has been shown to promote tumorigenesis in breast cancers, and several in vivo studies have shown that PTP1B knockout results in delayed tumour formation [129,133,134]. Inhibition of PTP1B results in decreased cell adhesion by downregulation of claudin-1, FAK, and E-cadherin and induces anoikis [120,135,136]. Recently, many anticancer agents have targeted for breast cancer in vitro and in vivo and have shown to act via inhibition of PTP1B activity. Some recent reports are presented in Table 2. Although, none of these studies report the direct involvement of PTP1B inhibition against cancer stem cells, there are a few reports in the literature suggesting the role of PTP1B in breast cancer stem cell specific pathways. The inhibition of PTP1B in D492 and HMLE cells induces loss of extracellular matrix attachment and apoptosis. The same report also suggested greater sensitivity of breast mesenchymal cells to PTP1B inhibitors, thereby signifying the role of PTP1B inhibitors for targeting cancer stem cell population in breast cancers [120]. Another possible mechanism of action of PTP1B inhibition against BCSCs involve the role of chemokine CCL5. CCL5 is secreted by the mesenchymal stem cells in breast cancer cells, which then enhances their motility, invasion, and migration [137]. PTP1B upregulates the expression of CCL5 in breast cancer cells; therefore, PTP1B inhibition can be a promising approach for the treatment of breast cancer [132].

### 5.3. PTP1B Inhibition of AMPK Activation: Can Vanadium Compounds Activate AMPK?

There is increasing evidence suggesting the link between PTP inhibition and AMPK activation. Several biochemical consequences of PTP inhibition are believed to be executed via AMPK activation [149]. PTP1B is known to negatively modulate AMPK in peripheral tissues [33,150,151], and PTP1B deficiency is known to cause AMPK activation and autophagy [152]. Many studies suggesting the development of novel PTP1B inhibitors for the management and treatment of diabetes also report enhancement of AMPK activity along with PTP1B inhibition [124,153,154]. Hence, there appears to be a clear link between PTP1B inhibition and the simultaneous AMPK activation.

This leads to an interesting hypothesis—can insulin mimetic vanadium compounds with PTP1B inhibitory activity also activate AMPK? This can have important implications in developing effective anticancer treatments, since vanadium compounds are also known for their anticancer action; however, their efficacy on cancer stem cells has not yet been explored in detail.

### 5.4. Vanadium-Mediated AMPK Activation—Reduced Adipogenesis

Obesity is commonly associated with diabetes, and many anti-diabetic drugs are known to inhibit adipocyte differentiation. The role of vanadium in AMPK activation has mainly been focussed on vanadium-mediated inhibition of adipogenesis. This arises as a direct consequence of the fact that adipocyte differentiation depends on several growth factors including IGF and insulin. Adipogenesis is the process by which mesenchymal stem cells differentiate and mature into adipocytes and is often considered a hallmark for cancer stem cells. It is a complex process involving the interplay of several proteins, such as PPARγ, and transforming growth factors, such as KLF and IGF. Vanadium compounds are known to inhibit adipogenesis and, thus, many insulin mimetic agents can also be used to treat obesity. The most generally accepted mechanism of vanadium-induced inhibition of adipocyte differentiation involves AMPK activation along with a few other pathways. Adipose tissues form the bulk of the breast cancer microenvironment. It is involved in the synthesis of various adipokines and signalling molecules (such as IL6, leptin, adipsin, and tumour necrosis factor-α) that play crucial roles in breast tumour formation and progression. Leptin signalling plays an important role in tumour progression and maintenance of the cancer stem cell population by upregulating the cancer stem cell signalling pathways, Notch and Wnt [155]. Similarly, IL6 also has implications in inducing epithelial–mesenchymal transitions and increasing the breast cancer stem cell population. Therefore, within the breast cancer microenvironment, adipocytes play a pivotal role in inducing and maintaining the cancer stem cell population, and inhibition of adipogenesis can prove to be a successful pathway for the treatment of breast cancer stem cells [156,157].

AMPK can undergo activation in the adipose tissue in response to stress such as malnutrition, exercising, and extreme cold. It can also be activated by endogenous molecules, such as high-density lipoproteins and IL6, in the adipose tissue including other activation pathways discussed above. Along with LKB1, AMPK in the adipose tissue can be phosphorylated at Thr^172^ by adipokines such as leptin and adiponectin. AMPK activation directly affects lipogenesis and fat accumulation. Activated AMPK phosphorylates several substrates resulting in the inhibition of the synthesis of fatty acids, triglycerides and cholesterol, and it promotes fatty acid oxidation.

**Figure 7:**
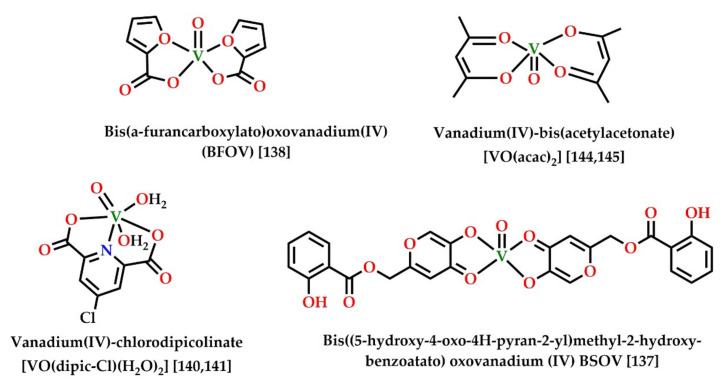
Structures of some vanadium (IV) complexes with reported AMPK activation activity.

The role and application of vanadium compounds in AMPK-mediated inhibition of adipogenesis are currently not very clearly understood. However, there are a few recent reports suggesting AMPK activation in insulin mimetic action as well as decreased adipogenesis by vanadium compounds [158,159,160]. Some recently reported vanadium compounds with significant adipogenesis inhibition activity are collected in Figure 7 and Table 3. Insulin mimetic vanadium compounds, such as VOSO_4_ and BMOV, can activate the GLUT translocator in adipose tissues [113]. Recently, a study reported the upregulation of hepatic AMPK levels and Akt cascade by a synergistic combination of metformin and bis(α-furancarboxylato)oxovanadium(IV) (BFOV) for the treatment of fatty liver disease [161]. Such reports of synergistic action of metformin and vanadium salts are not uncommon [162]. The most extensively studied vanadium compound in this context is vanadium(IV)chlorodipicolinate. It is known to inhibit adipogenesis in 3T3-L1 preadipocytes via LKB1-mediated activation of AMPK and the subsequent downregulation of ACC, FAS, FABP4, and PPARγ. It can also inhibit the storage of lipids and triglycerides in cells and upregulate microtube-associated light-chain proteins, inducing autophagy in hepatocytes by activation of the LKB1/AMPK pathway [163,164]. Vanadium-mediated insulin mimetic and anti-obesity action following AMPK action has also been reported using groundwater enriched with vanadium [165]. Similar results were also reported using vanadium extracted from *Brassica napus* cultured in vanadium-rich Jeju water. The vanadium-containing extract was reported to exhibit anti-diabetic effects as well as reduced triglyceride accumulation and adipogenesis in in vivo models. The suggested mechanism of action involved AMPK-mediated upregulation of glycogen synthesis by enhanced triacylglycerol lipase activity resulting in increased glucose uptake in adipocytes [166].

Vanadium catalysed AMPK activation and reduced adipocyte differentiation might also involve simultaneous activation of the peroxisome proliferator-activated receptor (PPARγ) [167,168,169]. PPARγ is a controller of metabolic pathways, and its activation is associated with the consequent activation of AMPK [160]. The anti-diabetic thiazolidinediones also exert their therapeutic effects by activating PPARγ. There are few reports suggesting the vanadium-mediated activation of PPARγ and AMPK [160].

Although the exact role of PPARγ in tumorigenesis is ambiguous, it can be modulated in a cell line specific manner to achieve anticancer properties [170]. PPARγ agonists have shown promising results in impeding cancer and reducing cancer stem cell population [171,172,173,174]. Cancer stem cells, particularly breast cancer stem cells, exhibit overexpression of inflammatory factors, such as NFκB, IL6 and IL8, that play a pivotal role in upregulating mammosphere regulatory genes (SLUG, Notch3 and Jagged1) and assist in the maintenance of the breast cancer stem cell population [175]. Upregulation of PPARγ negatively impacts on the cancer stem cell population by impeding the activity of these inflammatory growth factors. Thus, many PPARγ ligands and activators find potent anticancer applications, the most common being cisplatin.

Thus, insulin mimetic vanadium compounds inhibit PTP1B activity and activate AMPK and PPARγ to suppress adipogenesis. Additionally, vanadium compounds are generally redox active under physiological conditions and can readily interconvert between VO(IV) and VO(V) using Fenton-like reactions within the cell. This redox interconversion results in the generation of ROS. These ROS can, in turn, further cause PTP inhibition as well as induce cytotoxicity [114,176]. Consequently, the anticancer properties of oxidovanadium complexes are well documented in the literature [111,176,177,178]. Vanadium (IV) compounds have been shown to inhibit cell adhesion to the extracellular matrix and cell migration in osteosarcoma cells [179]. Notably, there are also a few reports suggesting the role of vanadium-mediated inhibition of TGFβ-induced EMT. Dysregulated EMT in cancer cells is attributed as one of the main causes of cancer metastasis and the generation of the mesenchymal cancer stem cell phenotype. The insulin mimetic activity of sodium vanadate results in downregulation of protein tyrosine phosphatase and the activated insulin receptor substrate -1 in A549 cells. The activated IRS-1 subsequently suppresses TGFβ-mediated EMT [180]. Similar results were also reported using vanadium(V)–peroxido–betaine complex on A549 cells in vitro and ex vivo. The study reported vanadium-mediated inhibition of TGFβ-induced EMT and disruption of mitochondrial membrane potential in tumour cells. Furthermore, the vanadium (V) drug was found to act synergistically with the clinically used drug carboplatin with reduced expression of cancer stem cell markers [181]. However, the role of vanadium in targeting breast cancer stem cells has yet not been explored, but there are many insulin mimetic vanadium compounds in clinical trials [109,182,183] that could have the potential of successfully treating breast cancer and cancer stem cells.

## 6. Conclusions

Non-oncology drugs have gained increasing attention recently for their possible anticancer action. These drugs with well-defined pharmacokinetic and pharmacodynamic profiles fast track into clinical trials. The most successful non-oncology drug for cancer so far is metformin. Metformin has shown potent activity against many cancers, particularly breast cancer and breast cancer stem cells. The drug exerts its anti-diabetic and anticancer action through AMPK activation. AMPK is an energy-sensing molecule playing pivotal roles in metabolic processes. The phosphorylation and activation of AMPK have several implications that result in reduced cell proliferation and apoptosis in cancer cells. Activated AMPK phosphorylates several downstream substrates resulting in downregulation of cyclins, cell cycle arrest, suppression of fatty acid, cholesterol, triglyceride syntheses, inhibition of the mTOR pathway and induction of autophagy and Warburg effect antagonism. All these factors are involved in breast cancer as well as breast cancer stem cells and, thus, there are ample reports suggesting the inhibition of one or more of these pathways causing anticancer activity. AMPK activation also has implications in impeding adipogenesis, and many vanadium-based insulin mimetic agents can inhibit adipocyte differentiation following AMPK activation. Vanadium-based insulin mimetic compounds are known PTP1B inhibitors, and few were/are in clinical trials for the treatment of type 2 diabetes and obesity. There is clear evidence of the link between the interplay of PTP1B inhibition and AMPK activation in the prognosis and treatment of breast cancer. Although the anticancer applications of vanadium compounds are well documented, not much has been reported regarding the action of vanadium on cancer stem cells. Vanadium compounds possess all the required abilities to target and inhibit cancer and cancer stem cells. The recent development of vanadium-catalysed suppression of adipogenesis and the vast history of vanadium-mediated PTP1B inhibition and ROS generation can be carefully manipulated to target cancer stem cells, particularly breast cancer stem cells. This review presents the possibility of employing vanadium-based PTP1B inhibitors for activation of AMPK and treatment of breast cancer by targeting their stem cell population.

## Figures and Tables

**Table 1:** Some recently reported compounds with anticancer action following AMPK activation.

Drug	Cell Lines	Activated AMPK-Mediated Action	Reference
FND-4b	MCF-7, T-47D, MDA-MB-231, HCC-1143, and HCC-1806 (CSCs) *	Downregulation of ACC, S6, and cyclin D1 activity	[56]
Metformin	EC109 and EC9706	Cell cycle arrest in G0/G1 phase	[65]
Simvastatin	HepG2 and Hep3B	G0/G1 arrest by upregulating p21	[66]
Marine sponge-derived smenospongine	MCF7, HBL100, and 16HBE (CSCs) *	Cell cycle arrest and downregulation of Nanog, Bmi1, and Sox2	[30]
Phenformin	MCF7, ZR-75-1, MDA-MB-231, and SUM1315	Downregulation of cyclin D1, cell cycle arrest at G1 phase, and downregulation of pERK in ER+ cells (MCF7 and ZR-75-1) only	[68]
Epigallocatechin gallate and analogues	MDA-MB-231 (CSCs) * Hep G2 and Hep 3B HCT116 and HT-29	Cell cycle arrest and downregulation of mTOR Downregulation of mTOR and lipogenesis Inhibition of lipogenesis and energy metabolism	[75,78,84]
Metformin	MCF-7 and MDA-MB-231 cells MIA PaCa-2 (CSCs) *	Downregulation of cyclin D1, cell cycle arrest at G1 phase, and suppression of mTOR	[87]
Metformin	RPMI8226 and U266	Induction of autophagy and G0/G1 cell cycle arrest and suppression of mTORC1 and mTORC2	[85]
Salinomycin	RB 383, WERI-Rb-1 and RB116 (CSCs) *	Inhibition of mitochondrial respiration and mTOR	[88]
AICAR	Glioblastoma, in vivo	Inhibition of lipogenesis and mTOR	[74]
MT 63-78	LNCaP, CL1, PC3, DU145, and HeLa	Inhibition of lipogenesis and mTOR	[77]
γ–Tocotrienol	MCF-7 and MDA-MB-231	Warburg effect	[104]
Baicalein	PC-3, DU145, and MDA-MB-231	Inhibition of mTOR and autophagy	[63]
Cyclovirobuxine D	MCF7	AMPK autophagy	[64]
Cucurbitacin E	HeLa, MCF7, and DU145	AMPK, autophagy, and reduced mTORC1	[105]

* CSCs: cancer stem cells.

**Table 2:** Some recently reported PTP1B inhibitors with promising anticancer activity against breast cancer.

Compound	Cell Lines	Action	Reference
Oleuropein	MCF7	Cytotoxicity	[134]
Curcumin and derivatives	MCF-7 and MDA-MB-231	ROS generation, cytotoxicity	[138]
Jamunones	MCF-7 and MDA-MB-231, TNBC	Downregulation of (PI3K)/Akt pathway-mediated apoptosis; G0/G1 phase arrest	[139]
Green tea catechins (epigallocatechin and epigallocatechin gallate)	MCF-7	Cytotoxicity	[140]
Alpha-lipoic acid (ALA) and its reduced form of dihydrolipoic acid (DHLA)	MCF7	PTP and SHP2 inhibition and cytotoxicity	[141]
Flavonoids from *Orthosiphon stamineus* Benth.	MCF7, MCF7/TAMR, and MDA-MB-231	Cytotoxicity	[142]
Docosahexaenoic acid	MCF7	Cytotoxicity	[143]
Oleanane triterpenes from *Camellia japonica*	MCF7, MCF7/ADR, and MDA-MB-231	Cytotoxicity	[144]
Curcumin and cinnamaldehyde	MCF 7	Cytotoxicity	[145]
Isoflavonoids from *Erythrina addisoniae*	MCF7, MCF7/ADR, and MDA-MB-231	Cytotoxicity	[146]
Pterocarpan derivatives from *Erythrina abyssinica*	*MCF7*, MCF7/TAMR, MCF7/ADR, and MDA-MB-231	Cytotoxicity	[147]
Aurintricarboxylic acid (Fe chelator)	MCF7	Inhibition of SHP2 phosphatases and cytotoxicity	[148]

**Table 3:** Vanadium compounds with reported AMPK phosphorylation and activation activity.

Compounds	Action	Reference
BFOV (BFOV + metformin)	Activation of AMPK and reduced hepatic steatosis	[161]
(VO(acac)_2_)	Activation of AMPK, p38, and PPARγ, stimulation of adiponectin	[167,168]
Vanadium protein complex	Activation of AMPK and LKB1 and decreased adipogenesis	[159]
(VO(dipic-Cl)(H_2_O)_2_)	Activation of AMPK/LKB1, autophagy and reduced lipid accumulation and adipogenesis	[163,164]
BSOV	Activation of AMPK and PPARγ Insulin mimetic action	[160]
Vanadium-containing Jeju groundwater	Activation of AMPK and reduced adipogenesis	[165]
